# Spinal tuberculosis treatment duration: Correlating MRI findings with therapeutic outcomes

**DOI:** 10.3205/dgkh000600

**Published:** 2025-11-28

**Authors:** Mahesh Shinde, Sangramsingh Dixit, Mihir Patel, Kshitij Sarwey, Sanket Jethlia, Shreyas Revankar, Deepanshu Garg, Rohan Bahl, Juily Satam, Samreen Chunawala, Yash Nav Singh, Arunojya Kumari, Ibrahim Ansari

**Affiliations:** 1 Department of Orthopaedics, HBT Medical College and Dr. RN Cooper Hospital Juhu, Mumbai, India; 2 Lalita Superspeciality Hospital, Guntur, India

**Keywords:** spine, tuberculosis, anti-tubercular therapy, therapy control, magnetic resonance imaging, tuberculosis, clinical recovery, neurologic deficits

## Abstract

**Introduction:**

Tuberculosis (TB) of the spine is the most common form of musculoskeletal TB, especially in developing countries, and may lead to serious complications if untreated. Early diagnosis, anti-tubercular therapy (ATT), and surgery when needed are key to effective management. Magnetic resonance imaging (MRI) is the most reliable tool for diagnosis, monitoring, and determining treatment duration. This study aims to evaluate the appropriate length of ATT and MRI’s role in guiding therapy in spinal TB.

**Methods:**

This 36-month prospective study included 100 spinal TB patients treated with standard ATT. Clinical, hematological, and MRI evaluations guided therapy duration. Biopsy confirmed diagnosis, and surgery was done when indicated. MRI healing was classified as complete, partial, or non-healed. ATT continued until MRI confirmed healing. Multidrug-resistant cases received second-line therapy.

**Results:**

In a cohort of 100 spinal tuberculosis patients (mean age 23.5 years), 70% were female, and 88% had category-1 TB, with 12% exhibiting multidrug resistance. The thoracic spine was most affected (64%), followed by lumbar (30%) and cervical (6%) regions.

Conservative treatment was administered to 78 patients, while 22 underwent surgery. Neurological deficits were present in 78% of cases. Diagnostic yields were highest with histopathology (50%), followed by GeneXpert (48%), smear microscopy (30%), and culture (28%). Inflammatory markers showed significant improvement: equivalent series resistance decreased from 34.95 to 13.1 mm/hr, and C-reactive protein from 32.4 to 6.3 mg/L over 12 months. MRI assessments revealed complete healing in 30% at 6 months and 80% at 12 months. By 18 months, recovery rates reached 89% clinically, 82% hematologically, and 88% radiologically.

**Conclusion:**

Clinical recovery occurs first, but MRI is the most reliable tool for determining antitubercular therapy duration in spinal TB due to its accuracy in assessing disease resolution.

## Zusammenfassung

**Hintergrund:**

Die Tuberkulose (TB) der Wirbelsäule ist die häufigste Form der muskuloskeletalen TB, vor allem in Entwicklungsländern. Sie kann unbehandelt zu schwerwiegenden Komplikationen führen. Eine frühzeitige Diagnose, eine antituberkulöse Therapie (ATT) und bei Bedarf eine Operation sind der Schlüssel für die wirksame Behandlung. Die Magnetresonanztomographie (MRT) ist das zuverlässigste Instrument für die Diagnose, die Überwachung und die Festlegung der Behandlungsdauer. Ziel der Studie ist es, die angemessene Dauer der ATT und die Rolle der MRT bei der Steuerung der Therapie bei spinaler TB zu bewerten.

**Methode:**

Die prospektive 36-monatige Studie umfasste 100 Patienten mit TB der Wirbelsäulen, die mit der Standard-ATT behandelt wurden. Klinische, hämatologische und MRT-Bewertungen bestimmten die Therapiedauer. Die Diagnose wurde bioptisch bestätigt. Bei Indikation wurde eine Operation durchgeführt. Im Ergebnis der MRT wurde die Heilung als vollständig, teilweise oder nicht geheilt eingestuft. Die ATT wurde fortgesetzt, bis die MRT die Heilung bestätigte. Bei Vorliegen multiresistenter Erreger wurde eine Zweitlinientherapie durchgeführt

**Ergebnisse:**

In der Kohorte der 100 Patienten mit Wirbelsäulentuberkulose betrug das Durchschnittsalter 23,5 Jahre. 70% waren weiblich. 88% hatten TB der Kategorie 1, wobei 12% eine Multiresistenz aufwiesen. Die Brustwirbelsäule war am häufigsten betroffen (64%), gefolgt von der Lendenwirbelsäule (30%) und der Halswirbelsäule (6%).

Bei 78 Patienten wurde eine konservative Behandlung durchgeführt, 22 wurden operiert. Neurologische Defizite traten in 78% der Fälle auf. Die höchste diagnostische Ausbeute lieferte die Histopathologie (50%), gefolgt von GeneXpert (48%), Abstrichmikroskopie (30%) und Kultur (28%). Die Entzündungsmarker zeigten eine deutliche Verbesserung. Die Erythrocytensedimentationsrate sank im Laufe von 12 Monaten von 34,95 auf 13,1 mm/h und das CRP von 32,4 auf 6,3 mg/L. MRT-Untersuchungen zeigten eine vollständige Heilung bei 30% nach 6 Monaten und bei 80% nach 12 Monaten. Nach 18 Monaten erreichten die Heilungsraten klinisch 89%, hämatologisch 82% und radiologisch 88%.

**Schlussfolgerung:**

Die klinische Genesung tritt zuerst ein, aber die MRT ist das zuverlässigste Instrument zur Bestimmung der Dauer der antituberkulösen Therapie bei spinaler TB, da sie das Abklingen der Krankheit genau beurteilen kann.

## Introduction

Tuberculosis is endemic in India, with the spine being the most common site for extrapulmonary tuberculosis, accounting for more than 50% of musculoskeletal TB cases [1]. In developing and underdeveloped countries, the disease often takes an aggressive course, particularly in children, adolescents, and young adults, leading to complications such as paraplegia, deformities, and abscess formation [2]. Patients with tuberculosis of the spine, if not treated, may experience serious complications secondary to cord compression, spinal artery thrombosis, deformities, and vertebral collapse, significantly impacting the affected population [3], [4].

A major challenge in treating spinal tuberculosis is poor compliance with the lengthy treatment regimen, which has contributed to the rise in multidrug-resistant (MDR) TB cases, further complicating management [5], [6]. Effective treatment of spinal TB requires early diagnosis, appropriate anti-tubercular therapy (ATT), and surgical intervention when indicated [7].

Spinal tuberculosis typically occurs due to hematogenous spread from other infected areas [8]. Diagnosis is based on clinical history, physical examination, hematological markers, the Mantoux test, and imaging techniques such as radiography and magnetic resonance imaging (MRI) [9]. Newer modalities such as PET scans and FDG-MRI are also being utilized [10]. Diagnosis can be challenging, as other conditions, such as bacterial or fungal infections and malignant diseases, may present with similar imaging findings. The gold standard for assessing suspected spinal tuberculosis is MRI, which provides a comprehensive visual representation of the disease’s extent and activity [11].

The most common form of spinal TB is paradiscal, although central, anterior, and posterior types are also observed [12]. Treatment generally includes a high-protein diet, antitubercular therapy (ATT), supportive care, and surgery when necessary [13]. Surgical intervention is indicated in cases of treatment failure, worsening neurological deficits, kyphotic deformity, or cold abscesses [14]. Standard procedures include debridement, decompression, fusion, and spinal instrumentation [15].

Modern anti-tubercular drugs have significantly improved recovery rates and reduced mortality, complications, and relapses. An important consideration in management is determining the appropriate duration of ATT [16]. Traditionally, surgeons prescribe fixed durations (e.g., 6, 9, or 12 months), but these are not always tailored to disease activity [17]. The optimal duration of spinal TB therapy is not universally agreed upon, with recommendations ranging from six to 18 months. Hence, the treatment duration for tuberculosis of the spine is based on clinical, radiological, and pathological findings rather than adhering strictly to set guidelines [7]. 

MRI remains the most effective tool for early diagnosis, assessing disease progression, and guiding treatment decisions. It is also the preferred modality for follow-up in both surgically and conservatively managed patients [18]. Serial MRI evaluations can reveal changes in vertebral bodies, intervertebral spaces, paraspinal abscesses, and kyphotic angles – factors that guide therapy and predict prognosis [19], [20]. 

The main goal of this study is to determine the length of antitubercular treatment for spine TB and the role MRI plays in deciding the total duration.

## Materials and methods

### Study procedure

This prospective observational study was conducted over 36 months and included 100 patients with spinal TB. All participants gave their informed consent, and the institution’s ethics committee approved the study. Patient details were documented using a standardized clinical history proforma at a tertiary care center.

Inclusion criteria were all age groups, diagnosed cases of spinal tuberculosis, and patient/guardian of minor patients who consented to take the complete ATT course as advised. Exclusion criteria were HIV/immunocompromised patients with spinal tuberculosis and patients who were not compliant with treatment.

**Figure 1:**
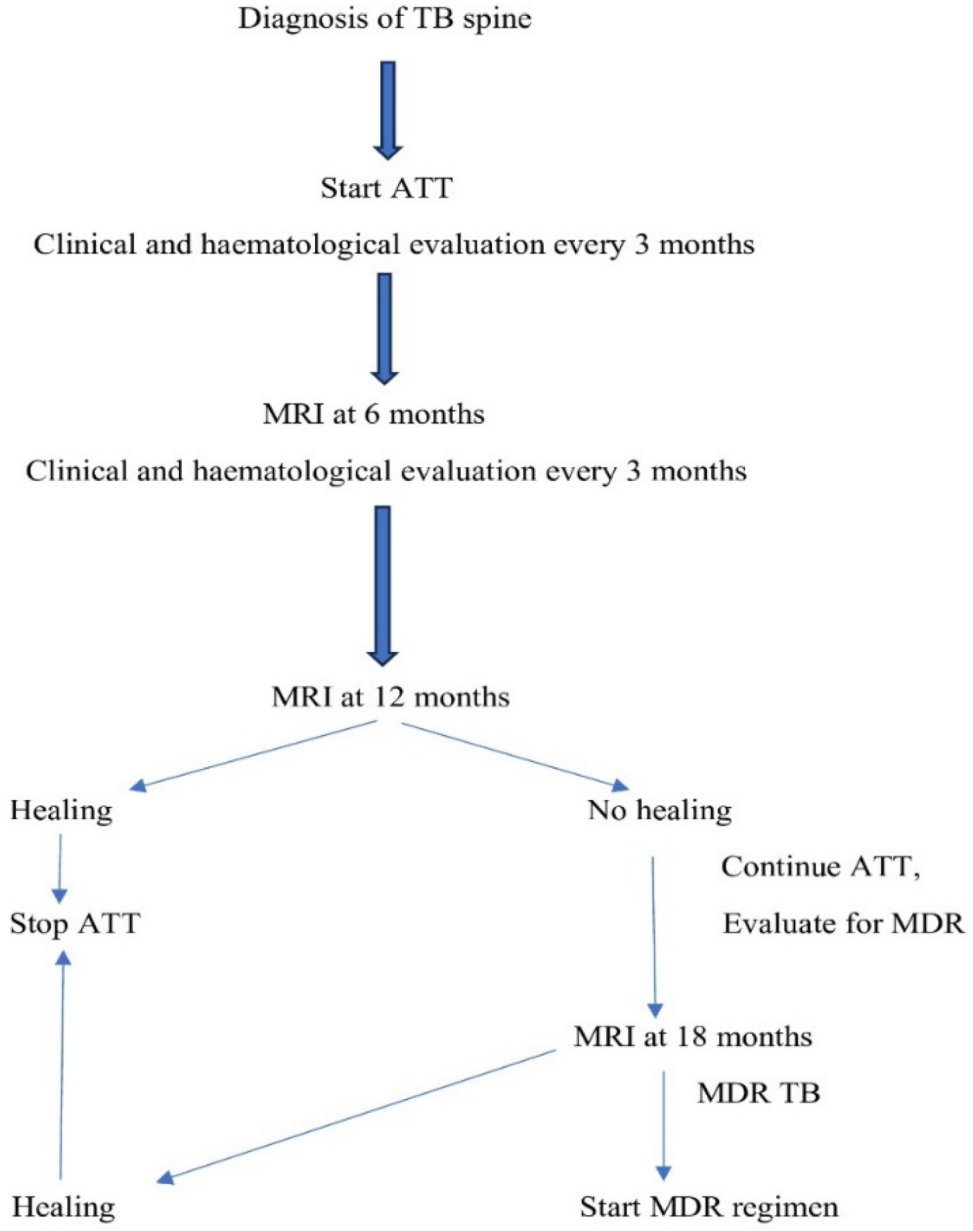
Study flowchart

After informed consent, all patients fulfilling the inclusion criteria were added to the study. Patients were evaluated by history, general status, systemic examination, and local examination. The hematological investigation included haemoglobin, differential leukocyte counts, liver function tests (LFT), renal function tests (RFT), Equivalent Series Resistance (ESR), and C-reactive protein (CRP). Radiological (x-ray) examination included chest radiographs, radiographs of the affected spine level, and MRI of the spine. CT-guided/fluoroscopic-guided biopsies was performed in all the patients, and the samples was sent for histopathology, mycobacteria growth indicator tube (MGIT), microscopy (Ziehl-Neelsen stain), and Gene Xpert. All patients who were diagnosed with spinal tuberculosis received consultationwith a thoracic medicine physician and started on antitubercular therapy. A middle-path regime followed with standard ATT, including a 2-month intensive phase (with isoniazid, rifampicin, ethambutol, and pyrazinamide), and a 10-month continuation phase (isoniazid and rifampicin) [21]. The treatment duration was extended in cases where clinical symptoms persisted and/or MRI or hematological findings indicated ongoing disease activity, even after 12 months of chemotherapy. Anti-tubercular therapy was continued until MRI confirmed complete healing and clinical parameters normalized. Those patients who had not shown any healing at 12 months after starting ATT were evaluated for multidrug resistance. In multidrug resistance cases, patients were further evaluated, and an MDR regimen was started with second-line antitubercular drugs (Fluoroquinolones, Bedaquiline, Linezolid, Clofazimine, Ethionamide, Aminoglycosides, and Cycloserine) for 18–24 months. Surgery was performed on the indicated patients, i.e., in cases of treatment failure, worsening neurological deficits, kyphotic deformity, or cold abscesses. The surgical procedures we performed included C-arm CT-guided biopsy, open biopsy, debridement, and posterior decompression and instrumentation. The study process is summarized in the following flowchart (Figure 1 (Fig. 1)).

### Outcome measures

The improvement was assessed clinically every month, with a hemogram with ESR and CRP at three monthly intervals and a MRI at 6 and 12 months. The clinical identification of a healed stage is based on the absence of all systemic disease activity features, local warmth, tenderness, spasm, cold abscess, sinusitis, and the return of painless motion (in early disease). Repeated erythrocyte sedimentation is normal or does not show a progressive increase in its value. The healing markers on radiolographs are considered to be bone outline repair, remineralization, the sharpness of cortical boundaries, and the repair of bony trabeculae. The healing in the MRI was classified into three types based on resolution [22], [23]: 

a. Completely healed or nearly healed cases:

- Resolution of marrow edema,
- reconstitution of previously destroyed bone; the marrow in the affected area may be replaced by fat after healing,
- complete resolution of paravertebral collections.

b. Partially healed cases:

- Resolution of marrow edema,
- fatty replacement of marrow,
- persistence of enhancement in the paravertebral collection.

c. Non-healed cases:

- No resolution of marrow edema,
- no fatty replacement of marrow,
- persistence of enhancement in the paravertebral collection.

After completion of treatment, patients were recalled every six months and assessed to find local recurrence of spinal TB. 

### Statistical analysis

The collected data were entered into a Microsoft Excel sheet and transferred to SPSS software ver. 22 for analysis. Qualitative data were presented as frequencies and percentages and analysed using the chi-square test. Statistical significance was set at P<0.05.

## Results

This study comprised 100 patients with spinal tuberculosis. The average age of the patients in the research population was 23.5 years. Just 30% of participants were men, whereas 70% of participants were women. Most patients (88%) were classified under category-1 TB (diagnosed case of tuberculosis without any resistance to antitubercular therapy), with 12% having multidrug-resistant (MDR) TB. The most often impacted spinal area was the thoracic (64%), followed by the lumbar (30%), and cervical (6%) regions. Conservative treatment was administered to 78 patients, whereas 22 underwent operative management. This distribution highlights the predominance of young females and thoracic involvement in spinal tuberculosis cases. Neurology was affected in 78 patients (Table 1 (Tab. 1)).

Smear microscopy was positive in 30% of cases, while culture positivity was slightly lower at 28%. Gene Xpert showed a higher detection rate, identifying Mycobacterium tuberculosis in 48% of patients. Histopathology had the highest positivity at 50%, suggesting its key role in diagnosis. These findings emphasize the importance of combining multiple diagnostic modalities for accurate detection, as reliance on a single method may lead to missed diagnoses (Table 2 (Tab. 2)).

Initially, before starting ATT, the mean ESR was 34.95 mm/hr, which steadily declined to 13.1 mm/hr at 12 months. Similarly, CRP levels experienced a substantial decline from a baseline of 32.4 mg/L to 6.3 mg/L after the 12 months. These decreasing trends in both ESR and CRP indicate a consistent improvement in the inflammatory status of patients during treatment. The reduction in values across follow-ups is clinically significant, reflecting a favourable response to ATT. A p-value <0.05 confirmed the statistical significance of this trend (Table 3 (Tab. 3)).

At 6 months, only 30% of patients showed complete resolution of the disease, while the majority (55%) demonstrated partial resolution, and 15% had no signs of healing. However, by 12 months, there was a significant improvement: 80% of patients showed complete resolution, 15% had partial healing, and only 5% showed no resolution. This indicates a marked progression in healing between the two time points. The improvement was statistically significant (p<0.05). These results highlight the importance of ATT (Table 4 (Tab. 4)).

Recovery rates improved over time, with clinical, hematological, and MRI-based healing reaching 89%, 82%, and 88%, respectively, by 18 months of ATT (Table 5 (Tab. 5)).

## Discussion

The ideal duration of treatment for spinal TB is debated due to the absence of a clear definition of “healed status”. While repeat histology would confirm cure, it is often not feasible. Several recommendations have been proposed. Clinical improvement and radiographic evidence of healing at the end of the prescribed ATT regimen without relapse at the 2-year follow-up were considered indicators of a healed condition [24]. Originally, Tuli et al. [25] described a “middle-path” regimen for spinal TB, which refers to a treatment approach that balances medical management (chemotherapy) with surgical intervention when necessary. Subsequently, a 6-month duration of ATT was suggested by the British Thoracic Society [26]. In their study, Upadhyay et al. [27] found that radiological outcomes were similar after 6, 9, and 18 months of treatment. The American Thoracic Society recommended a treatment of 6-9 months for tuberculosis of the spine [28]. Jain et al. [29] found that 60% of spinal TB patients showed MRI healing at 12 months of directly observed therapy (DOTS), and 90% had healed by 18 months. They emphasized the effectiveness of extended DOTS and recommended that ATT duration be guided by clinical, hematological, and contrast-MRI evaluation rather than fixed timelines. For bone and joint TB, the Index-TB guidelines [30] suggest an intense period of ATT lasting two months and a continuation phase lasting ten to sixteen months. Stopping treatment at six months is insufficient given the severity of spinal TB; the duration of therapy should be determined by clinical and radiological response rather than set deadlines [30].

Treatment of spinal TB involves a combination of ATT and surgical intervention in selected cases. A 2-month intense phase consisting of four medications (isoniazid, rifampicin, pyrazinamide, and ethambutol) is part of the typical ATT regimen. This is followed by a 10- to 12-month continuation period with isoniazid and rifampicin. The period may be extended in situations of drug-resistant tuberculosis or persistent symptoms. Surgery is indicated for patients with neurological deficits, spinal instability, severe deformity, or large abscesses, and commonly involves decompression, debridement, and spinal instrumentation.

Outcome measures include clinical improvement (pain relief, neurological recovery), hematological parameters (ESR, CRP), and radiological findings on MRI, such as the resolution of marrow edema and paravertebral abscesses. Follow-up MRIs at 6 and 12 months help assess healing, in addition to normalized inflammatory markers, neurological recovery, and radiological healing mark successful treatment. Early diagnosis, compliance with ATT, and timely surgical intervention contribute to favourable outcomes and reduced disability. Bhargava et al. [31] assessed the the healed status of spinal TB clinically (pain relief, symptom resolution, weight gain), hematologically (reduced ESR, improved hemoglobin, lymphocytosis), and radiologically. Radiographs showed vertebral healing signs; contrast-MRI confirmed resolution of abscesses and marrow edema. Only once MRI healing was established was ATT discontinued. If active disease persisted at 12 months, treatment continued with repeat imaging every 3 months until healing.

In our study, 88% of patients belonged to category 1 and received the standard regimen, while 12% of patients had multidrug-resistantTB and underwent the MDR regimen. The thoracic spine was most commonly affected (64%), followed by the lumbar (30%) and cervical (6%) regions. Ifthekar et al. [32] found the dorsal spine to be most commonly involved, followed by multifocal lesions. In their analysis of 356 implicated vertebrae, Sharma et al. [33] found that thoracic levels (T1-T10) were the most usually afflicted in 163 (45.78%), followed by thoracolumbar (T11-L2) vertebrae in 98 (27.52%) cases. Following the middle-path regimen, conservative treatment was given to 78 patients, and 22 underwent surgery. In a study by Abbas et al. [34] involving 37 patients, 9 underwent surgery, and 28 were managed conservatively.

In our study, histopathology yielded the highest positivity rate (50%), followed by Gene Xpert (48%), smear microscopy (30%), and culture (28%), highlighting the importance of using combined diagnostic approaches in spinal TB. Acharya et al. [35] also used a combined diagnostic approach, noting that CT-guided biopsy was most effective, while histopathology provided the earliest results. Pus culture, smear, and nucleic acid amplification test (NAAT) had low yields despite high sensitivity. In a study by Solanki et al. [36] of spinal TB cases, 62 out of 68 (91.2%) were Gene Xpert positive, 35 out of 64 (54.69%) tested positive in the acid-fast bacillus test, and 53 out of 60 (88.33%) had histopathological confirmation.

Over the course of one year, ESR and CRP levels steadily decreased, suggesting less inflammation and a favourable reaction to ATT. This trend was both clinically and statistically significant (p < 0.05). Jiang et al. [37] similarly found a significant decrease in ESR and CRP six and twelve months after commencing ATT. Similarly, Li et al. [38] reported a significant decline in ESR and CRP after ATT and surgery in neurologically affected spinal TB patients.

At 6 months in the present study, 30% of patients achieved complete resolution; this increased to 80% by 12 months. Partial and non-resolving cases declined from 55% and 15% to 15% and 5%, respectively. This significant improvement (p < 0.05) underscores the importance of sustained treatment and follow-up MRI to monitor healing and determine the duration of ATT. Le Page et al. [39] showed a healing rate of 40% at 6 months, which increased to 75% at 12 months on ATT.

In the present study, clinical recovery (42%) was higher than hematological (35%) and MRI-based recovery (30%) at 6 months. All indicators showed consistent healing improvement across modalities by 12 and 18 months, with clinical recovery leading the way (85%, 89%), followed by MRI (80%, 88%) and hematological recovery (76%, 82%). Singh et al. [40] showed healing in 46% of patients at 12 months, while 74% at 24 months showed suppression of signal intensity on short tau inversion recovery (STIR) images.

MRI is the most effective tool for early diagnosis, monitoring disease progression, and guiding treatment in spinal TB [41]. It is preferred for follow-up, as it reveals key changes that influence management and predict outcomes [19]. MRI plays a pivotal role in monitoring the healing status of patients with spinal TB during follow-up. It is highly sensitive in detecting early pathological changes such as bone marrow edema, abscess formation, and vertebral destruction, all of which are indicative of active infection. Serial MRI evaluations provide non-invasive insights into disease progression and recovery, aiding clinicians in tailoring individualized treatment plans. Completely healed or nearly healed cases show resolution of marrow edema, reconstitution of previously destroyed bone, fatty replacement of the marrow in the affected area, and complete resolution of paravertebral collections. Partially healed cases show resolution of marrow edema and fatty replacement of marrow, but persistent enhancement in paravertebral collections. In contrast, non-healed cases exhibit persistent marrow edema, absence of fatty marrow replacement, and continued enhancement in the paravertebral collection. These imaging findings are crucial for assessing the effectiveness of ATT and for guiding decisions regarding treatment duration. 

Jain et al. [29] used these criteria for MRI-based follow-up in spinal TB. They recommended that patients with non-healed but responding lesions after 8 months of treatment continue on alternate-day INH and rifampicin, with contrast MRI performed at 12 and 18 months until healed status is achieved. Similarly, Saini et al. [42] used the same MRI criteria during follow-up and stopped ATT only after complete healing was observed. In a study by Dwivedi et al. [43] on 49 consecutively managed spinal TB patients with or without neurological deficits over 1.5 years, the authors concluded that serial MRI scans are an essential modality for monitoring response to antitubercular therapy. Leowattana et al. [44] used a trifecta of clinical improvement, laboratory markers, and radiographic evaluation as corroborating indicators of healing status. They also showed that MRI often lags behind clinical recovery by up to 3 months.

## Conclusion

The study concludes that the duration of antitubercular therapy depends on clinical, hematological, and radiological recovery. Among these, clinical recovery shows the earliest response, while MRI shows a delayed response. However, MRI remains the most valuable and reliable indicator for determining the duration of antitubercular therapy in spinal tuberculosis.

## Notes

### Authors’ ORCIDs

- Shinde M: https://orcid.org/0000-0002-4091-9447
- Dixit S: https://orcid.org/0000-0002-2411-9108
- Patel M: https://orcid.org/0000-0001-6304-5845
- Sarwey K: https://orcid.org/0009-0000-3805-2445
- Jethlia S: https://orcid.org/0009-0009-2745-4241
- Revankar S: https://orcid.org/0009-0003-8072-4075
- Garg D: https://orcid.org/0009-0001-8085-8597
- Bahl R: https://orcid.org/0009-0002-8882-8760
- Satam J: https://orcid.org/0009-0008-7201-0714
- Chunawala S: https://orcid.org/0009-0002-8460-2131
- Singh YN: https://orcid.org/0009-0002-9422-500X
- Kumari A: https://orcid.org/0009-0005-1877-7816
- Ansari I: https://orcid.org/0009-0006-7805-3809

### Ethical approval 

Ethics committee approval taken from the institutional Ethics committee (registration no CDSCO-ECR/1654/ Inst/MH/2022)

### Funding

None. 

### Competing interests

The authors declare that they have no competing interests.

## Figures and Tables

**Table 1:**
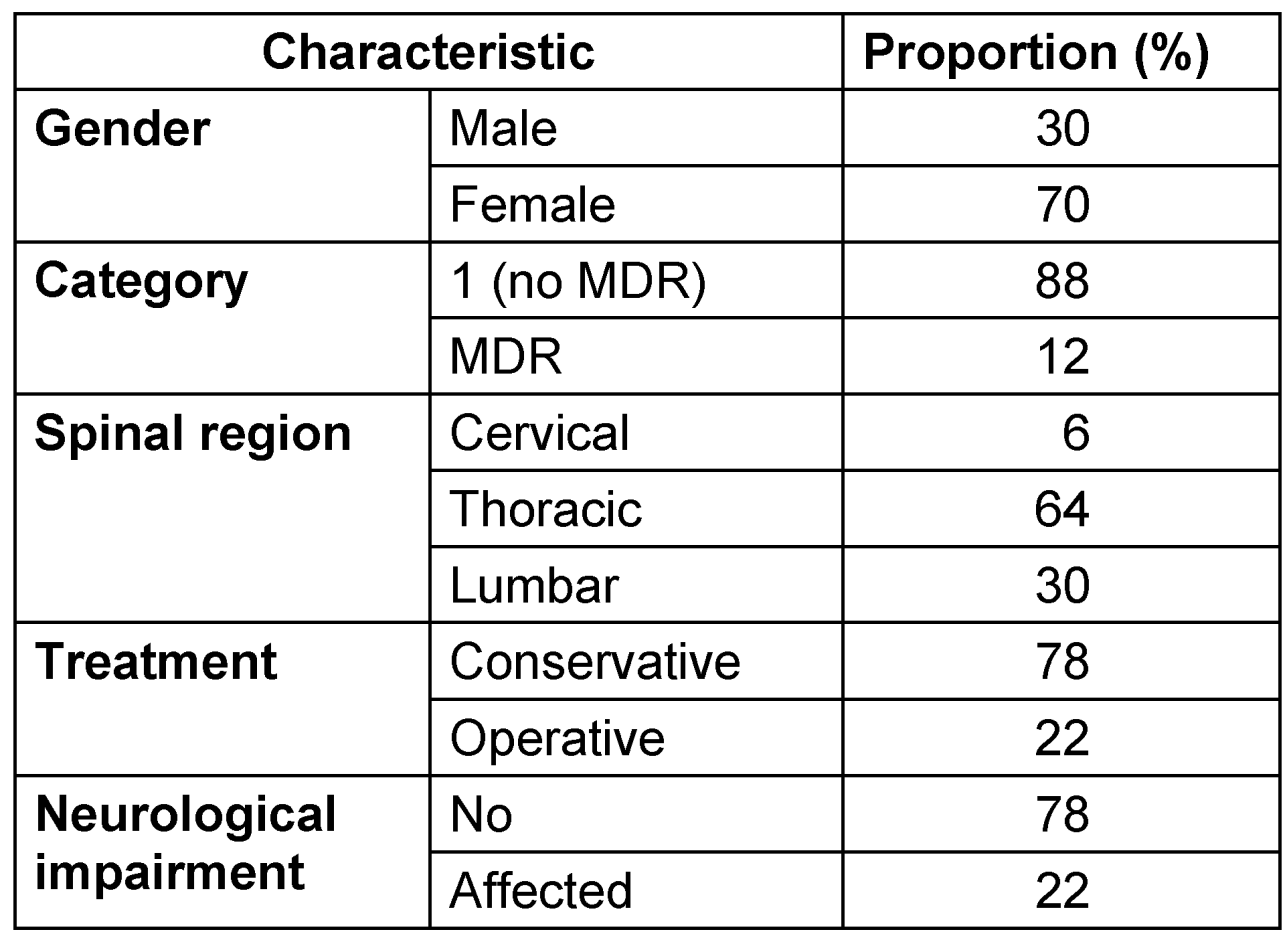
Characteristics of the studied cohort (n=100)

**Table 2:**
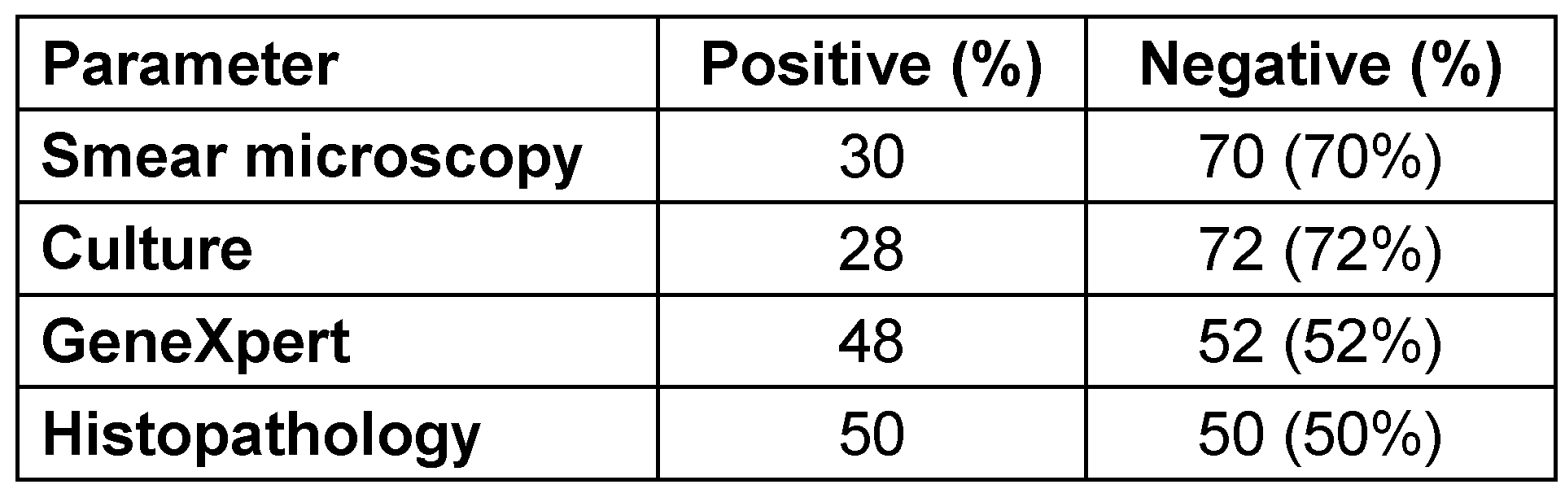
Microbiological and histopathological parameters of the studied cohort

**Table 3:**
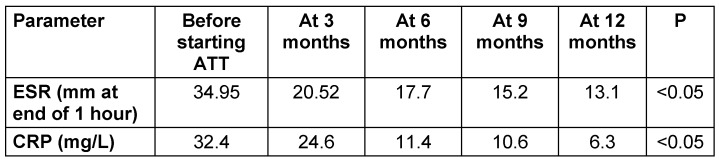
Hematological parameters of the studied cohort

**Table 4:**
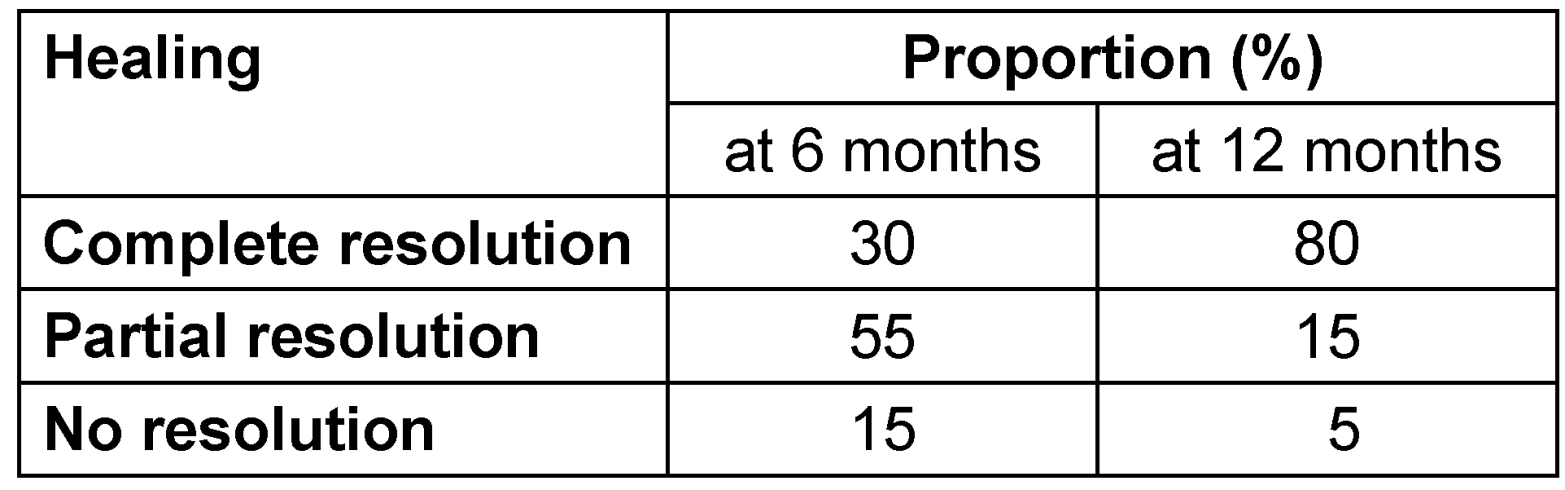
Magnetic resonance imaging (MRI) findings at follow-up

**Table 5:**
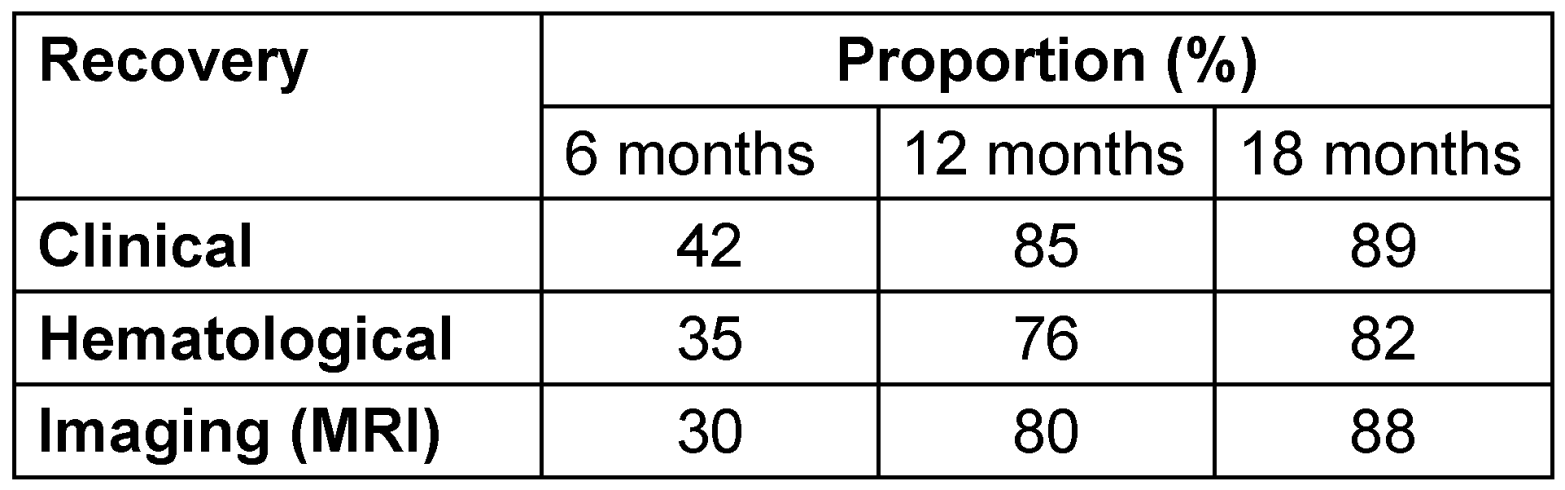
Development of outcome parameters
